# Prevalence of Vitamin D Inadequacy Among Chinese Postmenopausal Women: A Nationwide, Multicenter, Cross-Sectional Study

**DOI:** 10.3389/fendo.2018.00782

**Published:** 2019-01-07

**Authors:** Zhongjian Xie, Weibo Xia, Zhenlin Zhang, Wen Wu, Chunyan Lu, Shuqing Tao, Lijun Wu, Jiemei Gu, Julie Chandler, Senaka Peter, Hang Yuan, Ting Wu, Eryuan Liao

**Affiliations:** ^1^Department of Endocrinology and Metabolism, The Second Xiangya Hospital of Central South University, Changsha, China; ^2^Department of Endocrinology, Key Laboratory of Endocrinology, Peking Union Medical College Hospital, Chinese Academy of Medical Sciences, Beijing, China; ^3^Department of Osteoporosis and Bone Diseases, Shanghai Jiao Tong University Affiliated Sixth People's Hospital, Shanghai, China; ^4^Department of Endocrinology, Guangdong General Hospital, Guangzhou, China; ^5^Department of Endocrinology, West China Hospital, Sichuan University, Chengdu, China; ^6^Department of Orthopedics, The Second Affiliated Hospital of Harbin Medical University, Harbin, China; ^7^Department of Rheumatism and Immunology, People's Hospital of Xinjiang Uygur Autonomous Region, Urumqi, China; ^8^Department of Pharmacoepidemiology, Merck Research Laboratories, Merck & Co., Inc., Kenilworth, NJ, United States; ^9^Department of Medical Affairs, MSD (China) Co., Ltd., Shanghai, China; ^10^Asia Pacific Unit, Department of Pharmacoepidemiology, MSD (China) R&D Co., Ltd., Beijing, China

**Keywords:** vitamin D deficiency, postmenopausal women, winter, urban, intact parathyroid hormone

## Abstract

**Purpose:** We aimed to investigate the status of serum 25-hydroxyvitamin D [25(OH)D] among Chinese postmenopausal women in a multicenter cross-sectional study.

**Methods:** Non-institutionalized postmenopausal women aged ≥55 years were recruited from urban and rural areas in 7 geographically different regions in China. Subject enrollment was executed during the summer and the winter. Vitamin D insufficiency and deficiency were defined as 25(OH)D < 30 and< 20 ng/ml, and was measured by liquid chromatography-tandem mass spectrometry. Women were referred to a dual-energy x-ray absorptiometry (DXA) if they had a medium-to-high fracture risk suggested by Osteoporosis Self-Assessment Tool for Asians (OSTA).

**Results:** Among all subjects, 91.2% (1,535/1,684, 95%CI: 89.7, 92.5) had vitamin D insufficiency and 61.3% had vitamin D deficiency (1,033/1,684, 95%CI: 59.0, 63.7). The prevalence of vitamin D deficiency was significantly higher in urban dwellers (64.9 vs. 57.7% in rural, *P* = 0.002) and in winter-enrolled subjects (84.7 vs. 41.3% in summer, *P* < 0.0001). The prevalence of vitamin D inadequacy did not increase in trend by latitude and was numerically lower in women who had high fracture risk and osteoporosis. A non-curvilinear change of intact parathyroid hormone (iPTH) levels was observed at 25(OH)D >16.78 ng/mL.

**Conclusions:** The prevalence of vitamin D inadequacy was remarkable among Chinese postmenopausal women and independent of fracture risk assessed by OSTA or osteoporosis suggested by DXA. Winter season, urban residence, however not latitude, were significantly associated with a higher likelihood of vitamin D deficiency. Optimal vitamin D status for iPTH and bone-related outcomes merits further investigation in this population.

## Introduction

Vitamin D plays an important role in bone health by increasing intestinal absorption of calcium and phosphate and acts as a critical component in the regulation of bone turnover. Sunlight exposure is the primary source of vitamin D followed by dietary intake of vitamin D-rich or fortified foods, where available. Vitamin D deficiency, as measured by serum 25-hydroxyvitamin D [25(OH)D] (1), is associated with increased bone turnover, muscle weakness and falls, osteoporosis and fractures, and endocrine disorders including rickets in the young, osteomalacia in the elderly, and secondary hyperparathyroidism (2, 3). Inadequacy in vitamin D is a worldwide problem with unfavorable consequences, especially in elderly women (4–6). Global evidence has shown a considerable prevalence of vitamin D insufficiency among North American (6), European (7, 8), as well as Asian populations (9). However, vitamin D status of the population in Southeast Asian countries received relatively less attention. Despite efforts to determine the optimal or sufficient concentration of serum 25(OH)D, there is still no universal consensus on a definition of vitamin D deficiency or insufficiency (i.e., 25(OH)D < 30 or 20 ng/mL) as reflected in the recent disagreement between the Institute of Medicine (IOM) guidelines (10) and the recommendations made by the Endocrine Society (11).

Postmenopausal women are at high risk of vitamin D deficiency. Maintenance of serum 25(OH)D may protect this population from adverse skeletal outcomes (1). Observational studies in North and Northeast China (12–18) investigated vitamin D inadequacy across various urban populations. Different cutoff values were used to define vitamin D deficiency and variations in serum 25(OH)D measurement occurred due to non-standardized assay methods. Comparison of study results is somewhat difficult. In examining previous studies, epidemiological data focusing on Chinese postmenopausal women were insufficient. In addition, the current standardized laboratory method for measuring 25(OH)D by liquid chromatography-tandem mass spectrometry (LC-MS/MS) (19) was not universally used. Therefore, those studies may not have accurately estimated the prevalence of vitamin D inadequacy in postmenopausal women in China. Therefore, we aimed to describe the distribution of serum 25(OH)D levels among Chinese postmenopausal women who lived in both rural and urban areas by conducting a nationwide, multicenter, cross-sectional, epidemiological study. Secondly, risk factors for vitamin D deficiency and the relationship between 25(OH)D and intact parathyroid hormone (iPTH) were explored in this sampled population.

## Methods and Materials

### Study Design

This was a nationwide, multicenter, cross-sectional study to investigate the distribution of 25(OH)D levels among rural and urban-dwelling Chinese postmenopausal women from 7 geographic regions in China by different latitudes (from 45.75 to 23.17° north, Supplementary Figure 1). The selected regions represented a variety of geographic locations by latitude in China in order to assess the regional difference in serum 25(OH)D levels and risk factors for vitamin D deficiency. One tertiary hospital in each region was selected as the coordinating site based on the site's location (a major city in the region) and the investigator's medical specialty (endocrinologist, orthopaedist, or rheumatologist). Subject enrollment began in July 2013 and completed in February 2014. Considering the seasonal impact on sunlight exposure, a two-season enrollment strategy by summer vs. winter was executed for the study. The study sample size was evenly allocated across 7 sites where subjects were equally enrolled from the urban and rural areas, and from summer and winter seasons, respectively. MSD designed and sponsored the study and analyzed the data. The study was conducted in accordance with the guidelines of the International Conference on Harmonization and local regulatory guidance and was approved by independent ethics committees of all sites before the initiation of any study-related procedure.

### Subject and Enrollment

Women were eligible if they were Chinese aged 55 years or above and postmenopausal (defined as absence of menses either naturally or surgically for at least a year by self-reporting), and willing to comply study procedure as judged by the investigators; women were excluded if they were hospitalized or institutionalized (i.e., patient in long-term care or elderly care facility), had severe kidney disease under physician's care, had mentally or legally incapacitated, or other conditions that may preclude the completion of health-related questionnaire or informed consent process, or had participated in a study with an investigational medicinal product or device within 30 days prior to giving informed consent.

Subject enrollment was conducted in the summer (between July and September 2013) and the winter (between January and February 2014), respectively, in one calendar year for six geographic regions where seasons are distinct. A single enrollment period (between December 2013 and January 2014) was applied in the southern region (Guangdong) due to limited seasonal variation and relatively warmer climate. The investigators obtained a population list for potential participants from local rural committees or urban residents' committees and recruited subjects accordingly with the assistance from these committees via approved telephone contact, advertisements, or posters. For the recruitment of rural subjects, one representing area (one to two villages) was selected for community-based recruiting of women by approved posters and broadcasting. Women were then screened in an outpatient clinic in the coordinating site. Written informed consent was obtained from all subjects or their legal representatives before study screening.

### Clinical Assessment and Laboratory Measurement

A single study visit was arranged for the subject who was assessed as per study procedure onsite. Demographic data, medical history (non-active diseases/diagnoses) for the previous 5 years, and medication use (including vitamin D and calcium supplements) within the 4 weeks prior to the study visit were recorded for each subject. Height and weight were measured with shoes and heavy clothing removed using a standardized portable stadiometer and weighing scale. Fracture risk was assessed using the Osteoporosis Self-Assessment Tool for Asians (OSTA) (20), which has a demonstrated role in predicting fracture risks in Asian populations.

#### Subject Questionnaire

A structured, 30-item interviewer-administered questionnaire was used by the investigator onsite to assess potential factors influencing serum 25(OH)D levels including, general health, fall or fracture history, sun exposure (including time spent outside with and without sun protection and the body parts exposed), physical activity and daily, and weekly or monthly consumption of vitamin D-containing foods, such as eggs and fish. A sun exposure index was calculated using the reported number of hours per week spent outside without sun protection in the previous month multiplied by the percentage of the body exposed to sunlight (9% for the face, 1% for each hand, 9% for each arm, and 18% for each leg). Sun exposure of the chest, back, and abdomen were not included (21).

#### Laboratory Measurement

A fasting blood sample by 10 ml was collected from each subject and sent to a central laboratory (Quest Diagnostics, Shanghai, China). Serum 25(OH)D samples were measured using an API4000 (SCIEX™) LC-MS/MS, which quantified concentrations of 25(OH)D_2_ and 25(OH)D_3_ for the determination of total 25(OH)D. The limits of quantification (LOQs) for 25(OH)D_2_ and 25(OH)D_3_ were 2 and 3 ng/ml, respectively (LOQ total 25(OH)D = 3 ng/ml). The concentration of iPTH was measured using chemiluminescence (DPC Immulite 2000, SIEMENS) with an LOQ of 0.3 pg/mL. An additional fasting blood sample by 5 ml was collected from a subset of subjects enrolled from 3 regions (Beijing, Shanghai and Hunan) during the winter to measure bone turnover markers including serum C-terminal telopeptide of type I collagen (β-CTX) and serum N-aminoterminal propeptide of type I collagen (P1NP) using electrochemiluminescence (Roche E601 platform).

#### BMD Measurement

Bone mineral density (BMD) of the total hip, the lumbar spine, and the femoral neck were measured by dual-energy X-ray absorptiometry (DXA) with either a Hologic® or GE Lunar machine. Calibration with the manufacturer's phantom performed for routine clinical practice at each testing site was accepted.

### Objectives and Outcome Measures

The primary objective was to describe the distribution of serum 25(OH)D levels among postmenopausal women aged 55 years and older in different geographic regions of China overall and by the risk of fracture (low, medium or high) as assessed by OSTA. Secondary objectives were to examine the risk factors for vitamin D deficiency and to estimate the relationship between serum 25(OH)D and iPTH levels. An exploratory objective was set to estimate the correlation of serum 25(OH)D with bone turnover markers β-CTX and P1NP levels.

Vitamin D insufficiency was defined as serum 25(OH)D < 30 ng/mL; vitamin D deficiency was defined as serum 25(OH)D < 20 ng/ml and < 15 ng/mL (1, 3, 10). Osteoporosis was defined as a BMD T-score ≤ -2.5 at any anatomical site; osteopenia was defined as a BMD T-score between −2.5 and −1.0 (22). Fracture risk was stratified as low (>-1), medium (−1 to −4), or high (< -4) as calculated based on body weight and age formulated by OSTA (20).

### Sample Size and Statistical Analysis

Based on the literature (13–18), a prevalence estimate of 50% for vitamin D deficiency was used for sample size consideration. Assuming a 5% precision as expressed by a 95% confidence interval (CI) of the point estimate and a 10% discontinuation rate, a sample size of 424 postmenopausal women was needed. In order to evaluate residential (rural vs. urban) and seasonal (summer vs. winter) differences, the above sample size was quadrupled to a total of ~1,680.

Descriptive statistics were used to address the primary objective. Distribution of vitamin D levels was analyzed and presented categorically as the proportion and corresponding 95%CI of vitamin D inadequacy as defined and numerically as the mean ± SD (SE) for serum vitamin D levels among all subjects whose serum 25(OH)D were measured. The primary analysis was also performed in subjects stratified by fracture risk level as assessed by OSTA, and grouped by region, residence and season. A *post-hoc* Chi-Square test was used to compare the prevalence of low vitamin D status among subgroups whenever applicable. Univariate and multivariate logistic regression analyses were applied to identify risk factors for vitamin D deficiency. Results were represented as odds ratio (OR) with corresponding 95% CI and *P*-value. In multivariate logistic regression, variables including latitude, travel to the sunny area and walking outside were excluded due to collinearity with sunlight exposure. All selected variables from univariate analysis retained in the multivariate model presented by adjusted OR with corresponding 95%CI and *P*-value. In terms of the relationship between 25(OH)D and iPTH, Pearson's correlation coefficient was performed. Levels of iPTH were plotted against serum 25(OH)D to assess any relationship between the two values. A quadratic fit model with plateau was used to evaluate the association between serum 25(OH)D and PTH levels (18). In addition, the association between serum 25(OH)D and bone turnover markers ß-CTx and P1NP was explored by using univariate linear regression analysis. Clinical and biochemical variables including age, BMI, iPTH, and years since menopause were fit into the model. Analysis of variance (ANOVA) was used to compare the mean levels of β-CTx and P1NP in different subgroups categorized by serum 25(OH)D levels.

For demographics and clinical data, descriptive statistics were made to display the results. Chi-square or *t*-test was used to test the statistical significance for categorical or continuous variables wherever appropriate. There was no imputation for missing data in terms of 25(OH)D or other variables. All statistical analyses were performed using SAS 9.3 (SAS Institute, Cary, NC, USA) and a *P*-value of 0.05 was considered statistically significant unless otherwise specified.

## Results

### Subject Enrollment and Characteristics

**Figure 1:**
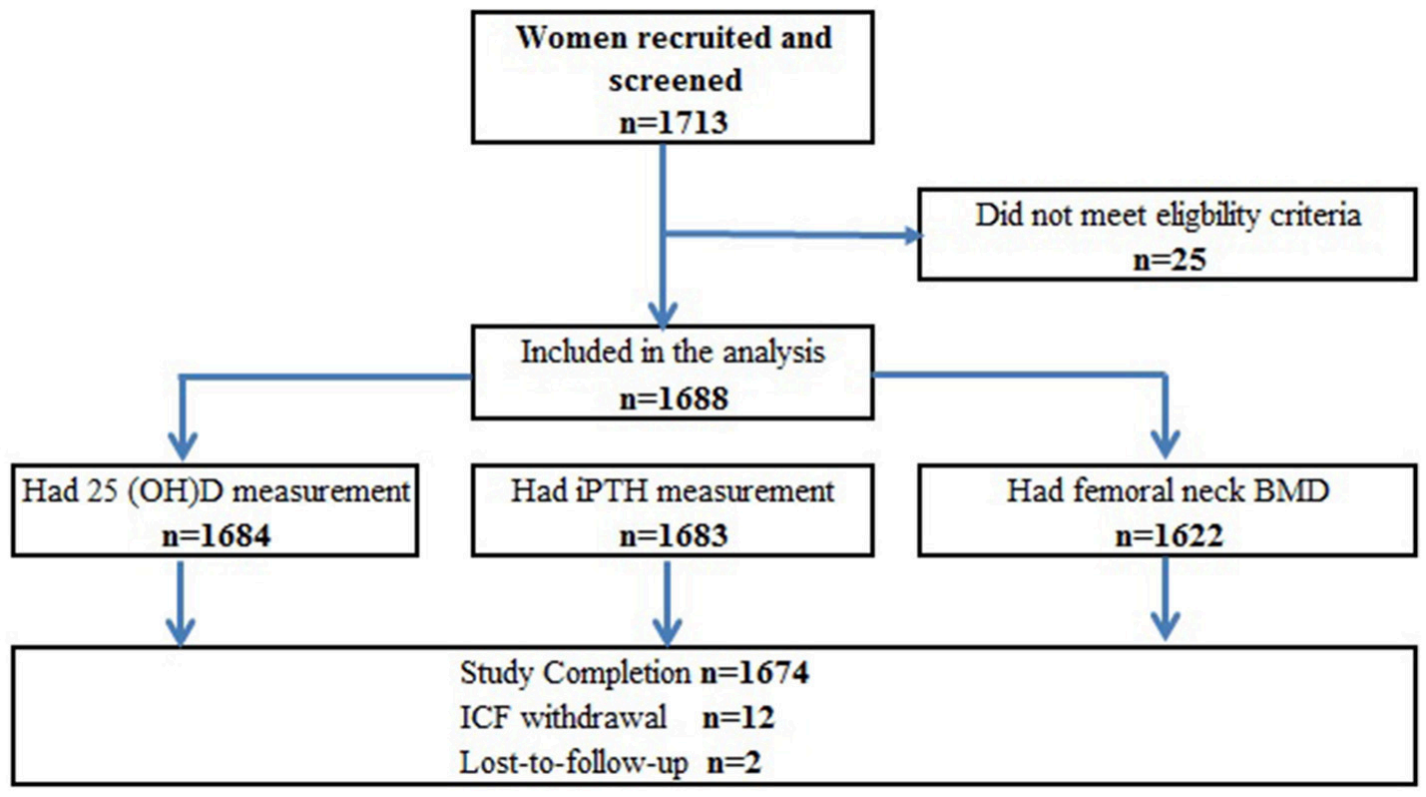
Study flowchart for enrolment. Four subjects had 25(OH)D < LLoQ and were not included in the analysis for the continuous variable. Subjects who had missing femoral neck DXA may have BMD measured at another anatomical site. Study completion was deemed as subjects who had all study procedures as per protocol. Subjects failed to return for the remained study procedure were deemed lost-to-follow-up. All subjects with non-missing data were included in the corresponding analysis.

The study recruited a total of 1,713 women from 7 regions in China, among which 25 women had screening failure and therefore, 1,688 subjects were included in the study analysis (Figure 1). Of those eligible, 1,684 postmenopausal women had 25(OH)D levels measured whereas four women did not complete the blood sampling procedure.

Results of the subject's characteristics are shown in Table 1. The subjects' mean (SD) age was 65.4 (7.6) years. Women who were elderly (>70 years) and obese accounted for 28.0, 3.6%, respectively, in the analyzed population. The proportion of women who had a high fracture risk as assessed by OSTA was 16.4% (276/1,688) in the analyzed population. There were 208 women (12.3%) reported fragility fractures, among which 25 (1.5%), 9 (0.5%), and 7 (0.4%) subjects had fractures at the spine, the pelvis, and the hip, respectively. Very few postmenopausal women used concomitant bisphosphonates [0.9% (9/1,688)] or calcium-containing supplements [3.6% (60/1,688)] in the study. Of all included subjects, 9.5% (160/1,688) had vitamin D supplementation and 30.9% (521/1,688) had high sunlight exposure. Subjects' mean serum calcium, phosphorus and PTH levels were within the normal ranges.

**Table 1:** Demographic and clinical characteristics for all included subjects.

	**Total**
**Characteristic**	**(*****N*** **=** **1,688)**
**Age (years)**	65.4 (7.6)
Elderly subjects (>70)	473 (28.0%)
**RESIDENTIAL REGION**	
Urban	848 (50.2%)
Rural	840 (49.8%)
**Body mass index (kg/m**^**2**^**)**	24.9 (3.6)
Obese (≥ 28.0)	311 (18.4%)
**LEVEL OF EDUCATION COMPLETED**	
No education	318 (18.8%)
Higher education	129 (7.6%)
**OSTA RISK INDEX**	
Low (>-1)	761 (45.1%)
Medium (−1 to −4)	651 (38.6%)
High (< -4)	276 (16.4%)
**FEMORAL NECK BMD T-SCORE**^**†**^	
Osteoporosis (T-score ≤ -2.5)	263 (15.6%)
**SELF-REPORTED MEDICAL HISTORY**	
Diabetes mellitus	204 (12.1%)
Osteoporosis	88 (5.2%)
Fragility fracture^∧^	208 (12.3%)
**Concomitant bisphosphonates use**	9 (0.5%)
**Calcium supplementation**^**¶**^	60 (3.6%)
**Vitamin D supplement use**^**§**^	160 (9.5%)
SUNLIGHT EXPOSURE^**^	
No	238 (14.1%)
High	521 (30.9%)
**Serum calcium (mg/dL)**	9.3 (0.4)
**Serum phosphorous (mg/dL)**	3.6 (0.4)
**Serum PTH (pg/mL)**	41.0 (23.0)

*Data are expressed as mean (SD) and number (%)*.

† *There are 62z subjects with missing femoral neck BMD values*.

∧ *There are 25 (1.5%), 9 (0.5%), and 7 (0.4%) subjects reported fractures at the spine, the pelvis, and the hip, respectively; 41 (2.4%) reported unspecified leg fractures*.

¶ *Includes calcium and calcium-containing supplements. Subjects who reported the use of more than one type of calcium supplement were only counted once*.

§ *Includes vitamin D and vitamin D-containing supplements. Subjects who reported the use of more than one type of vitamin D supplement were only counted once*.

** *Calculated as the number of hours per week spent outside without sun protection multiplied by percentage body part exposed to sunlight (9% for face, 1% for each hand, 9% for each arm, and 18% for each leg)*.

### Serum 25(OH)D Distribution and Vitamin D Inadequacy

Among all subjects with quantified 25(OH)D levels, the mean (SD) serum 25(OH)D was 18.0 (8.4) ng/ml. Mean serum 25(OH)D levels appeared comparable among geographic regions but were significantly higher in women who were enrolled in the summer as compared that in those who were enrolled in the winter within each region and overall (all *P* < 0.0001). A numerically higher mean serum 25(OH)D value was observed among rural residents compared to urban residents (mean difference ~1.3 ng/mL), which remained consistent across the regions, except for the Northwest and Southwest regions where higher mean serum 25(OH)D levels were observed in urban residents. Data on mean serum 25(OH)D levels by season and by residential region are displayed in Supplementary Table 1 and Supplementary Figure 2, respectively.

Table 2 presents the prevalence of vitamin D inadequacy as defined by different cutoff values overall and in subjects grouped by fracture risk assessed by OSTA and by osteoporosis measured by DXA. Vitamin D insufficiency, defined as 25(OH)D < 30 ng/mL, was 91.2% (1,535/1,684, 95%CI: 89.7, 92.5%) among all included subjects; vitamin D deficiency, defined as 25(OH)D < 20 or 15 ng/mL, was 61.3% (1,033/1,684, 95%CI: 59.6, 63.7%), 37.4% (630/1,684, 95%CI: 35.1, 39.8%), respectively, among all included subjects. Rates of vitamin D insufficiency and deficiency did not differ among various groups categorized by fracture risks or densitometric osteoporosis and were generally similar to those in the overall studied population.

**Table 2:** Prevalence of suboptimal vitamin D status.

**Population**	**Vitamin D insufficiency** **[25(OH)D < 30 ng/mL]**	**95% CI**	**Vitamin D deficiency** **[25(OH)D < 20 ng/mL]**	**95% CI**	**Vitamin D deficiency** **[25(OH)D < 15 ng/mL]**	**95% CI**
Overall	1,535/1,684 (91.2%)	(89.7, 92.5)	1,033/1,684 (61.3%)	(59.0, 63.7)	630 (37.4%)	(35.1, 39.8)
**FRACTURE RISK BY OSTA**						
Low (>-1)	706/759 (93.0%)	(91.0, 94.7)	479/759 (63.1%)	(59.6, 66.6)	302/759 (39.8%)	(36.3, 43.4)
Medium (−1 to −4)	586/649 (90.3%)	(87.8, 92.5)	391/649 (60.2%)	(56.4, 64.0)	233/649 (35.9%)	(32.2, 39.7)
High (< -4)	243/276 (88.0%)	(83.6, 91.6)	163/276 (59.1%)	(53.0, 64.9)	95/276 (34.4%)	(28.8, 40.4)
**FEMORAL NECK BMD**						
Normal	447/474 (94.3%)	(91.8, 96.2)	298/474 (62.9%)	(58.3, 67.2)	190/474 (40.1%)	(35.6, 44.7)
Osteopenia	805/889 (90.6%)	(88.4, 92.4)	544/889 (61.2%)	(57.9, 64.4)	324/889 (36.4%)	(33.3, 39.7)
Osteoporosis	232/263 (88.2%)	(83.7, 91.8)	150/263 (57.0%)	(50.8, 63.1)	90/263 (34.2%)	(28.5, 40.3)

*Data are presented as n/N (%)*.

*CI, Confidence interval*.

*Excluded 4 subjects with missing samples and include 4 subjects with serum 25(OH)D levels below LOQ (< 3 ng/mL)*.

**Figure 2:**
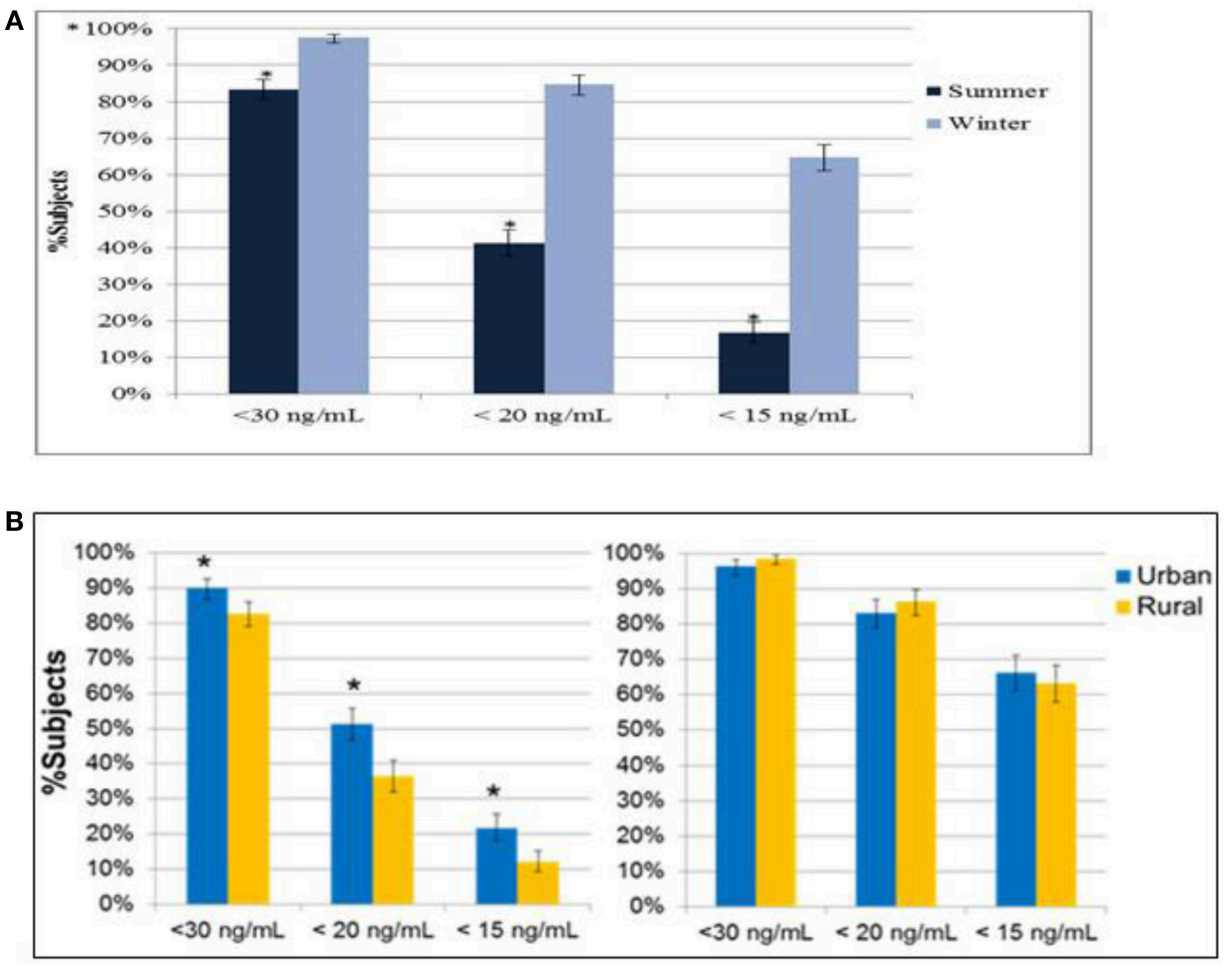
**(A)** Prevalence of vitamin D inadequacy by season. ^*^P < 0.01 from Chi-square test of the comparison of summer vs. winter subjects. **(B)** Prevalence of vitamin D inadequacy in urban and rural dwellers. ^*^P < 0.01 from Chi-square test of the comparison of urban vs. rural dwellers.

The prevalence of vitamin D insufficiency or deficiency was significantly lower in women who enrolled in the summer, compared to those who enrolled in the winter (all *P* < 0.01, 41.3 vs. 84.7% for serum 25(OH)D < 20 ng/mL, Figure 2A). A statistically significant difference in the prevalence of serum 25(OH)D inadequacy defined by different 25(OH)D cutoffs was seen between rural and urban dwellers enrolled in the summer (all *P* < 0.01, 46.8 vs. 35.7% for serum 25(OH)D < 20 ng/mL, Figure 2B). Cumulative distribution curves of serum 25(OH)D levels by region and season (summer and winter) are shown in Supplementary Figure 3. Regional variation in the prevalence of vitamin D inadequacy was more distinct in the summer than that in the winter.

### Risk Factors for Vitamin D Deficiency

The subject's demographic, lifestyle and clinical characteristics were analyzed individually to explore factors associated with vitamin D deficiency defined as serum 25(OH)D < 20 ng/ml by using univariate logistic regression (Supplementary Table 2). Overweight, region (Northwest, North, and Southwest), winter season, no exercise, no milk products or fish consumption, no vitamin D supplement, and no or low sunlight exposure were individual factors significantly associated with an increased risk of vitamin D deficiency (ORs between 1.28 and 7.90, all *P* < 0.05). Notably, a high fracture risk assessed by OSTA or densitometric osteoporosis was not associated with vitamin D deficiency.

A multivariate logistic regression model was established to explore the likelihood of vitamin D deficiency presented by adjusted OR by accommodating all variables analyzed in single logistic regression (Table 3). Variables with collinearity including latitude, travel to the sunny area and walking outside were excluded from the model analysis, as sunlight exposure was retained. Rural dwellers were less likely to have vitamin D deficiency (adjusted OR: 0.59, 95%CI: 0.40, 0.86, *P* < 0.01); women enrolled in the winter season had a 7.62-fold likelihood of having vitamin D deficiency as compared with those who were enrolled in the summer season (adjusted OR: 7.62, 95%CI: 5.52, 10.54, *P* < 0.0001); women with no vitamin D use had a 1.75-fold higher relative risk for vitamin D deficiency (adjusted OR: 1.75, 95%CI: 1.08. 2.85, *P* = 0.02); women who reported fair/poor health or no parental fractures had a relatively lower risk of vitamin D deficiency (*P* < 0.01). Sunlight exposure and fracture risk level by OSTA appeared to have no statistically significant association with vitamin D deficiency as adjusted for other variables in the model.

**Table 3:** Multivariate analysis of relative risk for vitamin D deficiency [25(OH)D < 20 ng/mL].

	**Total**	**% of subjects**	**Odds ratio**	**95% CI**	***P*-value**
	**(*****N*** **=** **1,684)**	**with 25(OH)D < 20 ng/mL**	**(Adjusted)**		
**AGE**					
≤70/>70	1,211/473	61.3/61.5	Ref/1.36	(0.89–2.10)	0.16
**RESIDENTIAL REGION**					
Urban/Rural	844/840	64.9/57.7	Ref/0.59	(0.40–0.86)	< 0.01
**EDUCATION**					
No education	317	65	Ref	–	
Primary/Elementary school	642	58.9	0.62	(0.42–0.93)	0.02
College/University/Graduate school	129	60.5	0.51	(0.25–1.02)	0.06
**BMI**					
< 24 kg/m^2^/≥24 kg/m^2^	702/982	58.0/63.7	Ref/1.11	(0.78–1.58)	0.57
SEASON^*^					
Summer/Winter	722/721	41.3/84.7	Ref/7.62	(5.52–10.54)	< 0.0001
**FRAGILITY FRACTURE AFTER 45**					
Yes/No	261/1,423	61.7/61.3	Ref/0.83	(0.56–1.23)	0.35
**GENERAL HEALTH**					
Excellent-Very Good/Fair-Poor	261/937	62.5/59.6	Ref/0.57	(0.38–0.86)	< 0.01
**READ OR TOLD ABOUT VITAMIN D AND BONE HEALTH**					
Yes/No	609/939	59.8/61.9	Ref/0.93	(0.65–1.32)	0.67
**FALL FROM STANDING HEIGHT**					
Yes/No	57/1,624	57.9/61.4	Ref/0.99	(0.46–2.15)	0.98
**PARENTAL HISTORY OF HIP FRACTURE**					
Yes/No	107/1,509	69.2/60.9	Ref/0.43	(0.23–0.81)	< 0.01
**ENGAGE IN STRENUOUS EXERCISE OR FARM WORKS**					
Yes/No	367/1,317	53.7/63.5	Ref/1.15	(0.82–1.62)	0.41
**TEA DRINK**					
Yes/No	615/1,069	61.3/61.4	Ref/0.83	(0.62–1.11)	0.21
**MILK PRODUCT WITH VIT D**					
Yes/No any milk product	56/919	48.2/61.7	Ref/1.41	(0.66–3.00)	0.38
FISH CONSUMPTION^**^					
Yes/No	708/976	65.8/58.1	Ref/0.86	(0.63–1.16)	0.32
**EGG WITH YOLK**					
Yes/No	1,256/428	58.9/68.5	Ref/1.60	(1.15–2.22)	< 0.01
**OSTA SCORE**					
Low (>-1)	759	63.1	Ref	–	
Medium (−1 to −4)	649	60.2	0.91	(0.63–1.32)	0.63
High (< -4)	276	59.1	0.82	(0.44–1.50)	0.51
**VITAMIN D SUPPLEMENT USE** ++					
Yes/No	150/1,534	50.7/62.4	Ref/1.75	(1.08–2.85)	0.02
SUN EXPOSURE INDEX^***^					
High	520	51.5	Ref	–	
Middle	457	66.7	1.31	(0.94–1.85)	0.12
Low	450	70.4	1.12	(0.79–1.60)	0.53

Subjects with “Unknown” in any category were excluded from analysis;

** Reported consumption of fish at least once in the past month;

*++Excluded subjects using active analogs (alfacalcidol and calcitriol)*.

*** *Calculated as the number of hours per week spent outside without sun protection multiplied by percentage body part exposed to sunlight (9% for face, 1% for each hand, 9% for each arm, and 18% for each leg). Sun exposure index was categorized into tertiles*.

### Relationship of Serum 25(OH)D to PTH, β-CTX, and P1NP

**Figure 3:**
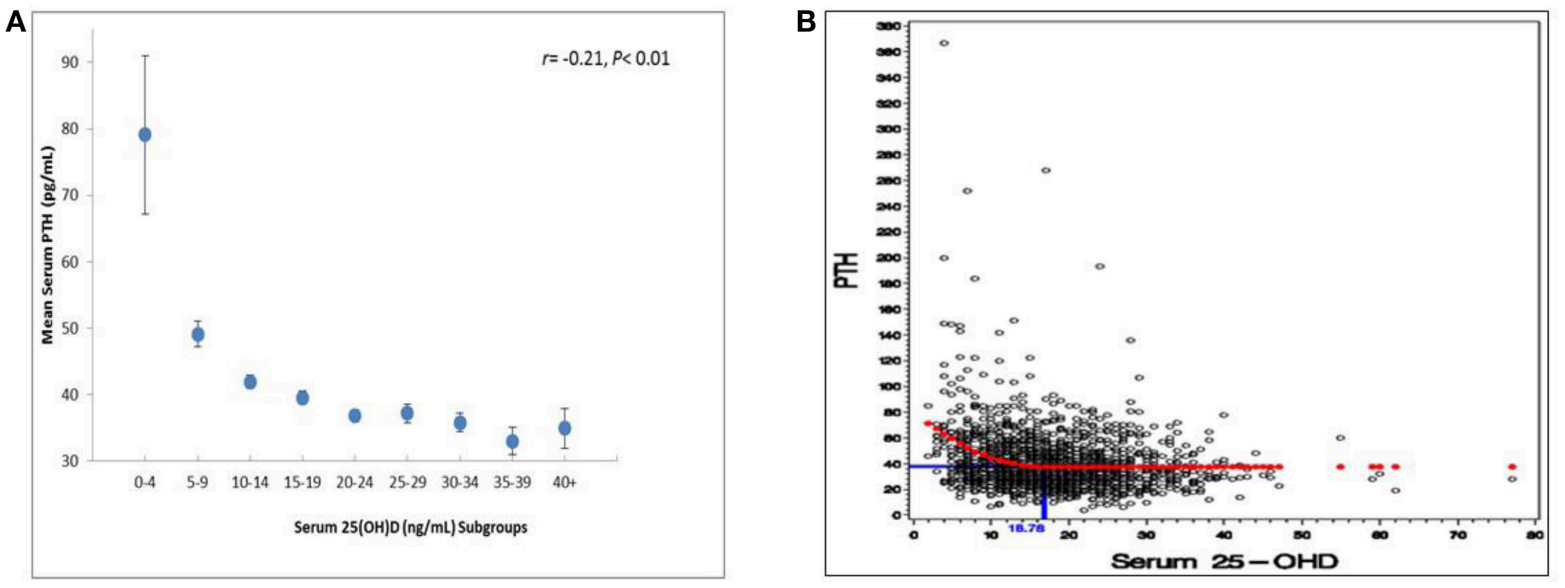
**(A)** Relationship between serum 25(OH)D and serum PTH levels (*N* = 1,679). Subjects with serum 25(OH)D levels >50 ng/ml were excluded (*n* = 4 summer and *n* = 1 winter). **(B)** Applied quadratic fit with plateau model to predict iPTH cutoff for 25(OH)D trends. Subjects with serum 25(OH)D levels >50 ng/ml were excluded (*n* = 4 summer and *n* = 1 winter).

Figure 3A suggested changes of iPTH levels over serum 25(OH)D intervals in 1679 subjects with quantified iPTH and 25(OH)D. A significant inverse correlation between serum iPTH and 25(OH)D was observed (*r* = −0.21, *p* < 0.01). The relationship between these two parameters was further analyzed by using a quadratic fit with plateau model (Figure 3B). Serum iPTH levels reached a plateau at a serum 25(OH)D level by 16.78 ng/ml, suggesting that the observed inverse relationship occurred below this cutoff value and iPTH remained stable for serum 25(OH)D above the cutoff.

A study subset of 360 women enrolled in the winter season from 3 geographic regions underwent the measurement of serum ß-CTx and serum P1NP. Univariate linear regression analysis showed that serum iPTH was significantly associated with serum ß-CTx (ß = 0.003, *P* = 0.04) but not P1NP (ß = 0.001, *P* = 0.59); BMI was inversely associated with serum ß-CTx (ß = −0.024, *P* < 0.0001) and P1NP (ß = −0.015, *P* < 0.01). In women grouped as 25(OH)D ≥30, 20–30, and < 20 ng/ml, respectively, neither ß-CTx nor P1NP differed significantly based on the analysis of variance (*P* = 0.22, *P* = 0.19), Table 4.

**Table 4:** Univariate linear regression analysis for serum ß-CTx and P1NP.

	**ß-CTx (ng/mL)** ***N*** **=** **360**			**P1NP (ng/mL)** ***N*** **=** **360**		
	**ß**	**SE**	***P*-value**	**ß**	**SE**	***P*-value**
25(OH)D, ng/ml	−0.004	0.004	0.25	−0.004	0.003	0.24
PTH, pg/ml	0.003	0.001	0.04	0.001	0.001	0.59
Age, years	−0.003	0.003	0.39	−0.002	0.003	0.42
YSM, years	−0.002	0.003	0.49	−0.001	0.002	0.55
BMI, kg/m^2^	−0.024	0.006	< 0.0001	−0.015	0.005	< 0.01

*Winter season sample from Beijing, Shanghai, and Hunan. SE, standard error; YSM, years since menopause **;** BMI, body mass index; ß-CTx, C-terminal telopeptide of type I collagen; P1NP, N-terminal propeptide of type I collagen.

*ß-CTx: 0.523 (0.22)/P1NP: 54.5 (24.0); data are presented as mean (SD); ß -CTx is log-transformed to achieve a normal distribution. ß-CTx and P1NP levels were not significantly different as grouped by subjects who had serum 25(OH)D> = 30, 20–30, and < 20 ng/mL, respectively (ANOVA P = 0.22, 0.19)*.

## Discussion

Findings from this multicenter, cross-sectional study in China suggested a considerable prevalence of vitamin D inadequacy among postmenopausal women from different geographic regions across the country. Although fracture risk level assessed by OSTA did not impact significantly on vitamin D status, urban dwelling, and the winter season contributed to lower 25(OH)D levels and were the associated risk factors on vitamin D deficiency among the analyzed population. In addition, our analyses did not show a curvilinear relationship between serum iPTH and serum 25(OH)D.

This study is among the few epidemiological studies to investigate serum 25(OH)D status using the standard LC-MS/MS assay in a large, geographically diverse population in China and Southeast Asia. The study compared serum 25(OH)D levels and prevalence rates in various scenarios including the season, residence location, and geographic region. The study well defined non-institutionalized postmenopausal women and obtained potential participant lists from the community. All blood samples were taken at the coordinating sites and sent to a central laboratory for further determination. Therefore, inter-laboratory variations were diminished and comparison with external study results can be realized and underestimation of 25(OH)D levels may be lessened (23).

Nevertheless, this study has limitations. The interview-based questionnaire caused the subject's recall bias on self-reporting events including fracture history and use of health supplements. DXA quality control was not done centrally which may cause measurement variations on BMD. Due to a cross-sectional design, a snapshot of the disease status at a certain time is given. The incident hypovitaminosis D over time cannot be assessed and causality cannot be inferred between suboptimal vitamin D and risk factors. The investigators recruited community and village women; however, a randomized sampling method was not performed to minimize selection bias. The study cannot be considered a strict population-based research and therefore, findings may not be generalizable to all postmenopausal women in China.

The prevalence of low serum 25(OH)D varies by region, population, and cutoff value used for hypovitaminosis D. Worldwide investigations (24) suggested prevalence of vitamin D deficiency [25(OH)D<20 ng/mL] was 8–57% in Caucasians and 2–70% in Southeast Asians across different age groups. Higher prevalence rates of hypovitaminosis D were seen in children, pregnant women and elderly people including postmenopausal women (24), or if 25(OH)D<30 ng/mL is used as cutoff (1). Among postmenopausal Caucasians, a study reported that hypovitaminosis D [defined 25(OH)D < 30 ng/mL, < 20 ng/mL] was 52, 18%, respectively, in North American elderly women who received concomitant bisphosphonates (21). A mean serum 25(OH)D of 30.4 ng/ml was observed. Numerically higher prevalence rates were found in Europe. In a large study on 8,532 postmenopausal subjects, the prevalence of 25(OH)D inadequacy (defined as 25(OH)D < 50 ng/mL, < 80 ng/mL) was 32.1 and 79.6%, respectively (25), and a mean serum 25(OH)D level of 27.2 ng/mL was observed. In a systematic review of 36 published studies (26), the prevalence of vitamin D deficiency [serum 25(OH) < 20 ng/ml)] ranged from 1.6 to 86% among community-living and institutionalized postmenopausal women, and a higher prevalence was seen in women with osteoporosis (12.5–76%) or with a history of fracture (50–70%) by using lower cutoff values in defining hypovitaminosis D. Our study, however, suggested a considerably higher prevalence of suboptimal vitamin D among Chinese postmenopausal women [25(OH)D < 30 ng/mL: 91.2%, 25(OH)D < 20 ng/mL: 61.3%], which was supported by a few published studies from East Asian regions (89.7, 72.1% in Beijing and central south China, and 65.0% in Korea] (13, 16, 27), and South Asia (>70% across different age groups in India) (28). It is difficult to directly compare attributable socioeconomic and lifestyle factors among populations. Differences in hypovitaminosis D in Caucasians and Chinese may be explained at the study level, including recruitment strategy, population sampling or assay method. As a low proportion (9.5%) of women in our study had vitamin D supplementation, hypovitaminosis D would be unsurprisingly frequent. Based on observational studies and randomized trials (1, 29), a serum 25(OH)D level of 20 ng/mL was shown to protect most people against bone-related events such as fractures and falls. Screening vitamin D deficiency and prescribing vitamin D supplements need more clinical attention in Chinese postmenopausal women.

Vitamin D is critical for bone mineralization. There has so far been no robust evidence on skeletal benefits associated with 25(OH)D levels based on pooled analyses on observational studies (30, 31). Moreover, the association of vitamin D with BMD and osteoporosis remains controversial. Several studies have shown that 25(OH)D is positively correlated with BMD (32–35) when 25(OH)D levels are low (36), whereas no such an association has been found in other studies (37–39). In our study, vitamin D inadequacy was not associated with higher fracture risk as assessed by OSTA or densitometric osteoporosis. Further, there was no statistically significant difference in bone turnover markers including ß-CTx and P1NP among a subset of subjects grouped by 25(OH)D interval from three geographic regions. Results must be interpreted with caution as very few women had bisphosphonates in addition to a low proportion of vitamin D or calcium supplementation. Another interesting finding is the lack of correlation between vitamin D insufficiency and fragility fracture prevalence. The subject's self-reporting on fragile fractures was prone to bias and thus, limited the precision in the analysis of association between these two morbidities. The prevalence of fragile fractures was low and major fragile fractures were very few, compared with previous reports (40). Results might not be generalizable even if this small cohort had more vitamin D supplementation or markedly lower vitamin D levels than those among most women in the study. For risk factors on fragile fractures in Chinese postmenopausal women, no sufficient evidence has been generated so far. Our study focused on risk factors for hypovitaminosis, but some results might be interpreted to link the lower prevalence of major fragile fractures. We recruited women who were relatively younger and had higher BMI, lower proportion of high OSTA or osteoporosis, and infrequent falls. These clinically meaningful factors have an association with fragile fractures, as supported by treatment guidelines (41, 42). Our study did not analyse the association between fragile fractures and fracture risk assessment (BMD or OSTA). However, results suggested an observed parallel trend among these clinical parameters. A lower proportion of women with densitometric osteoporosis or OSTA high risk was consistent with fewer fragile fractures. Findings indicated the primary role of BMD or OSTA in the screening of patients with high fracture risks among Chinese postmenopausal women, albeit further evidence needed.

Vitamin D levels may be affected by a number of factors including age, cultural behavior, latitude and season, and outdoor activity (24). Our subgroup analyses suggested the prevalence of hypovitaminosis D was significantly higher in women enrolled in the winter, and in women living in the urban community, respectively. Adjusted for all variables in the multivariate logistic model, urban dwelling, winter season, parental history of hip fracture, no consumption of eggs with yolks, and lack of vitamin D supplement was found to have a significant association with vitamin D deficiency among postmenopausal women. The study did not include smoking as a lifestyle factor in the study analysis, because we considered a very low proportion of subjects who smoke. Influence of occupation on vitamin D levels were measured as “engage in strenuous exercise or farm works” and “sun exposure index” as potential risk factors in the multivariate logistic model. As expected, season and residence location were major factors affecting vitamin D status. Latitude, however, was not a substantial influence. No prevalence gradient of vitamin D deficiency by latitude was observed, although rates varied by region (ranging from 50 to 71.3%). Similarly, one recent population-based study on 33 Chinese healthy adults suggested vitamin D deficiency was independent of latitude changes (43). These results suggested that there might be factors other than distance to the equator affect vitamin D status among Chinese postmenopausal women.

Our data indicate that the relationship between iPTH and 25(OH)D is not curvilinear, similar to previous studies. An inverse correlation between these two parameters was seen but iPTH levels remained stable in women who had 25(OH)D at 16.78 ng/mL or above. Historically, the normal lower limit of 25(OH)D was set at 30 ng/mL because PTH levels rise as 25(OH)D falls below this threshold, along with optimal calcium absorption (1, 44). In addition, the rationale for such a threshold extended to extra-skeletal benefits including cancer prevention (45). Recent osteoporosis guidelines have also suggested the optimum level of 25(OH)D is 30 ng/mL or above (41, 42). On the contrary, the IOM indicated that 20 ng/mL is appropriate for at least 97.5% of population (16 ng/mL for 50% of population) to maintain bone health as regulated by calcium and PTH status (46). A most recent trial has demonstrated ineffectiveness of maintaining vitamin D 30 mg/mL to cancer prevention in women >55 years of age (47). In the present study, the 25(OH)D threshold for such a rationale was 16.78 ng/mL, although calcium absorption was not possible to measure. A few studies in Chinese (48) and African (49) populations revealed very similar vitamin D levels (17–19 ng/mL) for PTH stability. The existing literature brings concerns the current cut-off for vitamin D deficiency among Chinese postmenopausal women. In terms of 25(OH)D < 20 ng/mL, a majority of women were vitamin D deficient and thus the use of vitamin D supplements was far from satisfactory, which is similar to other countries or regions. However, the proportion of vitamin D deficiency was dramatically lower when 25(OH)D < 15 ng/mL was used for the definition (61.3 vs. 37.4%). Although there is no consensus, there may be a reason to tune down 25(OH)D levels in the clinical setting. Future endeavors can be made to confirm optimal vitamin D status managed by diet intake and nutrient supplementation in relation to skeletal outcomes for this indicated population.

In conclusion, the prevalence of vitamin D inadequacy was remarkable among Chinese postmenopausal women and was independent of fracture risk assessed by OSTA or osteoporosis suggested by DXA. Winter season, urban residence, however not latitude, were significantly associated with a higher likelihood of vitamin D deficiency. Optimal vitamin D status for iPTH and bone-related outcomes merits further investigation in this population.

## Author Contributions

ZX, WX, ST, JG, JC, SP, TW, and EL conceived and designed research. ZX, WX, WW, ZZ, CL, LW, TW, and EL collected data and conducted research. ZX, WX, ZZ, CL, JC, SP, and TW analyzed and interpreted data. ZX, WW, ST, SP, and TW wrote the initial paper. ZX, WX, CL, LW, JG, JC, SP, HY, and EL revised the paper. EL had primary responsibility for final content. All authors read and approved the final manuscript.

### Conflict of Interest Statement

JC, SP, HY, and TW were employees of MSD at the time of study conduct and analysis. The remaining authors declare that the research was conducted in the absence of any commercial or financial relationships that could be construed as a potential conflict of interest. The reviewer XY declared a shared affiliation, with no collaboration, with one of the authors CL to the handling editor.

## Supplementary Material

Supplementary Table 1 Mean serum 25(OH)D (ng/mL) by overall, region and season.

Supplementary Table 2 Univariate analysis on relative risk for vitamin D deficiency (< 20 ng/mL).

Supplementary Figure 1 Distribution of enrolled subjects by province and region. ^*^Subjects were enrolled from urban and rural areas in seven provinces located in seven different geographic regions of China.

Supplementary Figure 2 Mean serum 25(OH)D levels by residential region. Rural residents had a higher mean serum 25(OH)D level as compared with urban residents, which remained consistent across the regions, except for the Northwest and Southwest regions where higher mean serum 25(OH)D levels were observed in urban residents.

Supplementary Figure 3 Cumulative distribution for serum 25(OH)D overall and by region. Cumulative distribution curves of serum 25(OH)D levels by region and season (summer and winter) are displayed. Regional variation in the distribution of serum 25(OH)D was more distinct in the summer compared to the winter when the distributions for all regions were more similar to the overall distribution. Lower serum 25(OH)D was observed in the North and Southwest regions in both the summer and winter.
