# Supplementary material for: Prevalence of Vitamin D Inadequacy Among Chinese Postmenopausal Women: A Nationwide, Multicenter, Cross-Sectional Study
Source: Front Endocrinol (Lausanne). 2019 Jan 7;9:782. doi: 10.3389/fendo.2018.00782 (PMC6330713; doi:10.3389/fendo.2018.00782)
Supplement: Supplementary Table 1 — Mean serum 25(OH)D (ng/mL) by overall, region and season. [file Table_1.doc]

**Supplementary Table 1 Mean Serum 25(OH)D (ng/mL) by Overall, Region and Season**

| **Region** | **Province** | **Approximate Latitude** | **25(OH)D (ng/mL)** | **Mean (SE)** | **25th - 75th Percentile** | **Summer Season** | **Winter Season** | ***P* value^†^** |
| --- | --- | --- | --- | --- | --- | --- | --- | --- |
|  |  |  | **N (Summer/Winter)** |  |  | **Mean (SE)** | **Mean (SE)** |  |
| **North East** | Heilongjiang | 45.75 N | 240 (120/120) | 18.5 (0.65) | 11.0 - 25.0 | 24.7 (0.81) | 12.4 (0.64) | <.0001 |
| **North West** | Xinjiang | 43.77 N | 237 (120/117) | 16.6 (0.58) | 10.0 - 22.0 | 20.1 (0.70) | 13.0 (0.80) | <.0001 |
| **North** | Beijing | 39.92 N | 240 (120/120) | 15.7 (0.42) | 10.5 - 21.0 | 19.6 (0.46) | 11.8 (0.50) | <.0001 |
| **East** | Shanghai | 34.50 N | 240 (120/120) | 18.6 (0.51) | 13.0 - 22.0 | 21.4 (0.68) | 15.8 (0.66) | <.0001 |
| **South West** | Sichuan | 30.67 N | 240 (120/120) | 15.3 (0.42) | 10.0 - 20.5 | 19.0 (0.54) | 11.7 (0.44) | <.0001 |
| **Middle** | HuNan | 28.22 N | 242 (122/120) | 21.2 (0.65) | 13.0 - 28.0 | 28.0 (0.82) | 14.4 (0.50) | <.0001 |
| **South** | Guangdong | 23.17 N | 241 (241) | 19.7 (0.37) | 16.0 - 24.0 | - | - | - |
|  | OVERALL |  | 1680 (722/717)(241) | 18.0 (0.20) | 12.0 - 23.0 | 22.2 (0.30) | 13.2 (0.25) | <.0001 |
| ^†^ SE=Standard error. 25(OH)D = 25-hydroxyvitamin D; | | | | | | | |  |
| The location in the South (Guangdong/Guangzhou) enrolled subjects from December 2013 to January 2014 and was defined as non-seasonal, and excluded from analysis for comparison. | | | | | | | |  |
| Excluded 4 subjects with missing samples, and 4 subjects with 25(OH)D below LOQ. †Comparison between seasons was performed using Student’s t test. | | | | | | | |  |
