# Supplementary material for: Prevalence of Vitamin D Inadequacy Among Chinese Postmenopausal Women: A Nationwide, Multicenter, Cross-Sectional Study
Source: Front Endocrinol (Lausanne). 2019 Jan 7;9:782. doi: 10.3389/fendo.2018.00782 (PMC6330713; doi:10.3389/fendo.2018.00782)
Supplement: Supplementary Table 2 — Univariate analysis on relative risk for vitamin D deficiency (< 20 ng/mL). [file Table_2.doc]

**Supplementary Table 2 Univariate Analysis on Relative Risk for Vitamin D Deficiency (<20 ng/mL)**

|  | **Total** | **% of subjects** | **Odds Ratio** | **95% CI** | **P-value** |
| --- | --- | --- | --- | --- | --- |
|  | **(N =1684)** | **with 25(OH)D <20 ng/mL** | **(Unadjusted)** |
| **Age** |  |  |  |  |  |
| ≤ 70 | 1211 | 61.3 | ref | - | - |
| > 70 | 473 | 61.5 | 1.011 | (0.812 - 1.257) | 0.9244 |
| **Residential Region** |  |  |  |  |  |
| Urban | 844 | 64.9 | ref | - | - |
| Rural | 840 | 57.7 | 0.738 | (0.606 - 0.898) | 0.0025 |
| **Education** |  |  |  |  |  |
| No Education | 317 | 65 | ref | - | - |
| Primary/Elementary School | 642 | 58.9 | 0.772 | (0.584 - 1.020) | 0.0686 |
| High School/Secondary School | 455 | 64 | 0.956 | (0.708 - 1.290) | 0.7692 |
| Trade/Technical/Non-University | 141 | 56.7 | 0.707 | (0.471 - 1.060) | 0.0931 |
| College/University/Graduate School | 129 | 60.5 | 0.824 | (0.541 - 1.256) | 0.3686 |
| **BMI** |  |  |  |  |  |
| < 24 kg/m^2^ | 702 | 58 | ref | - | - |
| ≥ 24 kg/m^2^ | 982 | 63.7 | 1.275 | (1.045 - 1.554) | 0.0166 |
| **Latitude:** |  |  |  |  |  |
| 45.75 N (Northeast) | 240 | 55.4 | ref | - | - |
| 43.77 N (Northwest) | 241 | 68 | 1.713 | (1.182 - 2.485) | 0.0045 |
| 39.92 N (North) | 240 | 70.4 | 1.915 | (1.314 - 2.790) | 0.0007 |
| 34.50 N (East) | 240 | 62.9 | 1.365 | (0.947 - 1.967) | 0.095 |
| 30.67 N (Southwest) | 240 | 71.3 | 1.993 | (1.366 - 2.909) | 0.0003 |
| 28.22 N (Middle) | 242 | 50 | 0.805 | (0.562 - 1.151) | 0.2339 |
| 23.17 N (South) | 241 | 51.5 | 0.853 | (0.596 - 1.220) | 0.3836 |
| **Season*** |  |  |  |  |  |
| Summer | 722 | 41.3 | ref | - | - |
| Winter | 721 | 84.7 | 7.902 | (6.146 - 10.160) | <.0001 |
| **Fragility fracture after 45** |  |  |  |  |  |
| Yes | 261 | 61.7 | ref | - | - |
| No | 1423 | 61.3 | 0.983 | (0.749 - 1.289) | 0.9014 |
| **General health** |  |  |  |  |  |
| Excellent/Very Good | 261 | 62.5 | ref | - | - |
| Good | 486 | 64.2 | 1.078 | (0.789 - 1.472) | 0.6365 |
| Fair/Poor | 937 | 59.6 | 0.885 | (0.667 - 1.174) | 0.3974 |
| **Read or Told about Vitamin D and bone health** |  |  |  |  |  |
| Yes | 609 | 59.8 | ref | - | - |
| No | 939 | 61.9 | 1.092 | (0.887 - 1.346) | 0.4069 |
| **Fall from standing height** |  |  |  |  |  |
| Yes | 57 | 57.9 | ref | - | - |
| No | 1624 | 61.4 | 1.156 | (0.677 - 1.975) | 0.5945 |
| **Parental history of hip fracture** |  |  |  |  |  |
| Yes | 107 | 69.2 | ref | - | - |
| No | 1509 | 60.9 | 0.695 | (0.455 - 1.061) | 0.0914 |
| **Travel to sunny area** |  |  |  |  |  |
| Yes | 112 | 68.8 | ref | - | - |
| No | 1572 | 60.8 | 0.705 | (0.467 - 1.065) | 0.0971 |
| **Walking outside** |  |  |  |  |  |
| Yes | 1164 | 61 | ref | - | - |
| No | 520 | 62.1 | 1.048 | (0.847 - 1.297) | 0.6638 |
| **Engage in strenuous exercise or farm works** |  |  |  |  |  |
| Yes | 367 | 53.7 | ref | - | - |
| No | 1317 | 63.5 | 1.5 | (1.187 - 1.895) | 0.0007 |
| **Tea drink** |  |  |  |  |  |
| Yes | 615 | 61.3 | ref | - | - |
| No | 1069 | 61.4 | 1.003 | (0.818 - 1.229) | 0.979 |
| **Milk product with Vit D** |  |  |  |  |  |
| Yes | 56 | 48.2 | ref | - | - |
| Milk product without Vit D | 473 | 61.7 | 1.733 | (0.994 - 3.021) | 0.0526 |
| No any milk product | 919 | 61.7 | 1.73 | (1.007 - 2.971) | 0.0469 |
| **Fish consumption**** |  |  |  |  |  |
| Yes | 708 | 65.8 | ref | - | - |
| No | 976 | 58.1 | 0.72 | (0.589 - 0.880) | 0.0013 |
| **Egg with yolk** |  |  |  |  |  |
| Yes | 1256 | 58.9 | ref | - | - |
| No | 428 | 68.5 | 1.513 | (1.199 - 1.910) | 0.0005 |
| **OSTA score** |  |  |  |  |  |
| Low (>-1) | 759 | 63.1 | ref | - | - |
| Medium (-1 to -4) | 649 | 60.2 | 0.886 | (0.714 - 1.099) | 0.2705 |
| High (<-4) | 276 | 59.1 | 0.843 | (0.636 - 1.117) | 0.2352 |
| **Femoral Neck BMD T-score** |  |  |  |  |  |
| Normal (T-score ≥ -1) | 474 | 62.9 | ref | - | - |
| Osteopenia (-2.5 < T-score < -1) | 889 | 61.2 | 0.931 | (0.740 - 1.172) | 0.5441 |
| Osteoporosis (T-score ≤ -2.5) | 263 | 57 | 0.784 | (0.577 - 1.066) | 0.1204 |
| **Vitamin D supplement use++** |  |  |  |  |  |
| Yes | 150 | 50.7 | ref | - | - |
| No | 1534 | 62.4 | 1.615 | (1.154 - 2.261) | 0.0052 |
| **Sun exposure index***** |  |  |  |  |  |
| High | 520 | 51.5 | ref | - | - |
| Middle | 457 | 66.7 | 1.887 | (1.455 - 2.446) | <.0001 |
| Low | 450 | 70.4 | 2.241 | (1.718 - 2.923) | <.0001 |
| **Years since Menopause, years** | 1672 |  | 1 | (0.989 - 1.011) | 0.9788 |
| Subjects with “Unknown” in any category were excluded from analysis | | | | | |
| *Includes subjects enrolled from 6 locations in Summer (July - September 2013) and Winter (January - February 2014). | | | | | |
| **Reported consumption of fish at least once in the past month | | | | | |
| ++Excluded subjects using active analogues (alfacalcitrol and calcitriol). | | | | | |
| ***Calculated as number of hours per week spent outside without sun protection multiplied by percentage body part exposed to sunlight (9% for face, 1% for each hand, 9% for each arm and 18% for each leg). Sun exposure index was categorized into tertiles. | | | | | |
