# Supplementary figures and images for: Prevalence of Vitamin D Inadequacy Among Chinese Postmenopausal Women: A Nationwide, Multicenter, Cross-Sectional Study
Source: Front Endocrinol (Lausanne). 2019 Jan 7;9:782. doi: 10.3389/fendo.2018.00782 (PMC6330713; doi:10.3389/fendo.2018.00782)

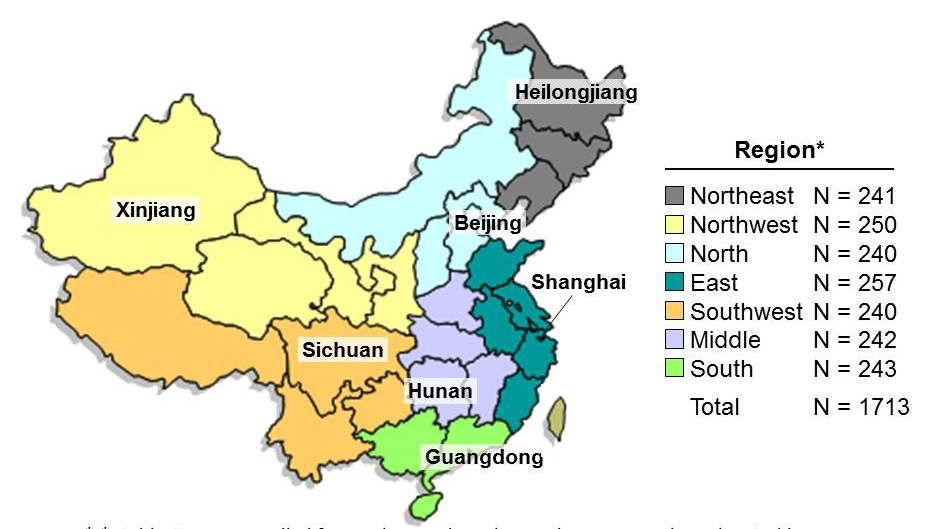

Supplement: Supplementary Figure 1 — Distribution of enrolled subjects by province and region. *Subjects were enrolled from urban and rural areas in seven provinces located in seven different geographic regions of China. [file Image_1.TIF]

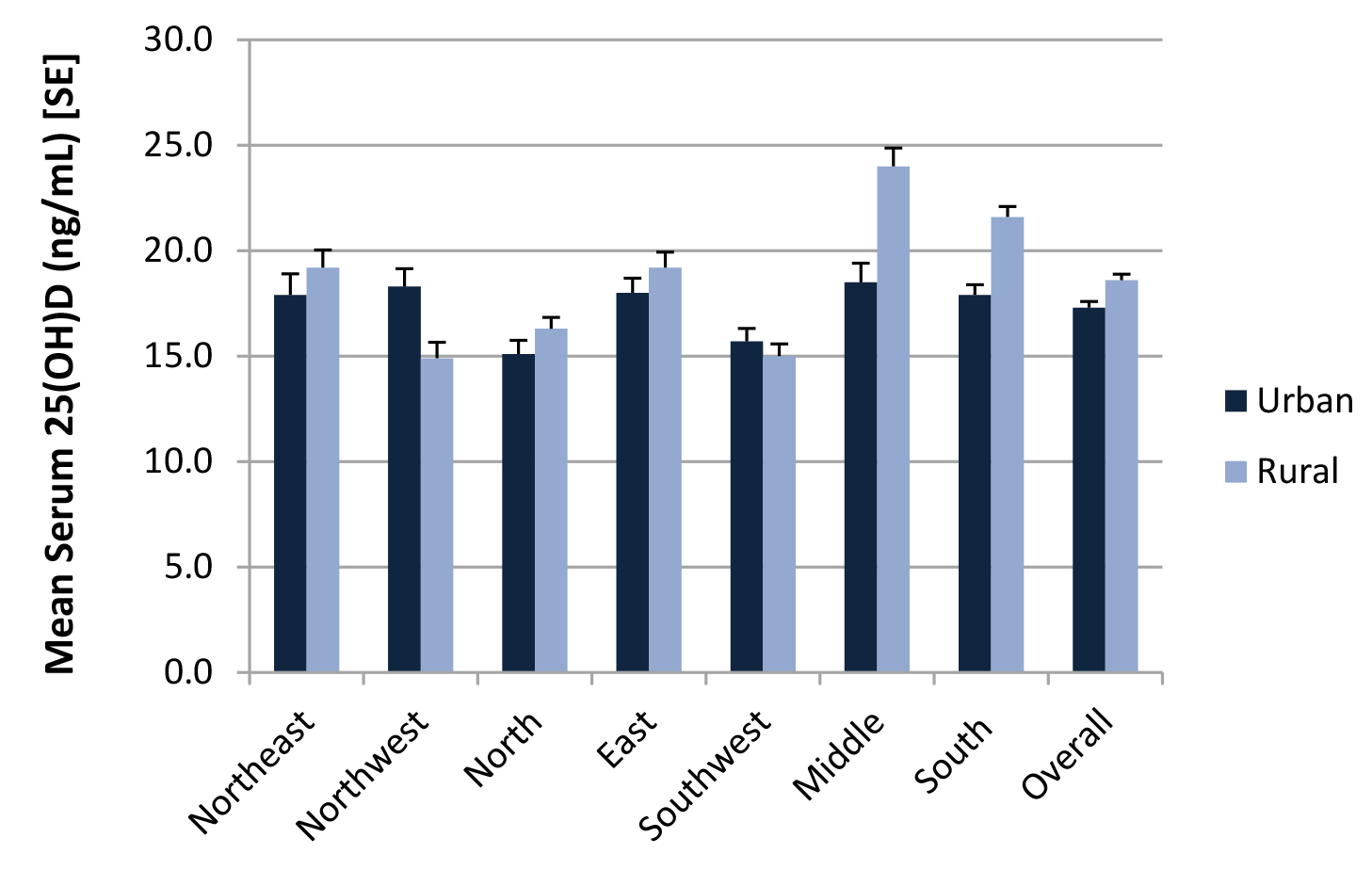

Supplement: Supplementary Figure 2 — Mean serum 25(OH)D levels by residential region. Rural residents had a higher mean serum 25(OH)D level as compared with urban residents, which remained consistent across the regions, except for the Northwest and Southwest regions where higher mean serum 25(OH)D levels were observed in urban residents. [file Image_2.TIF]

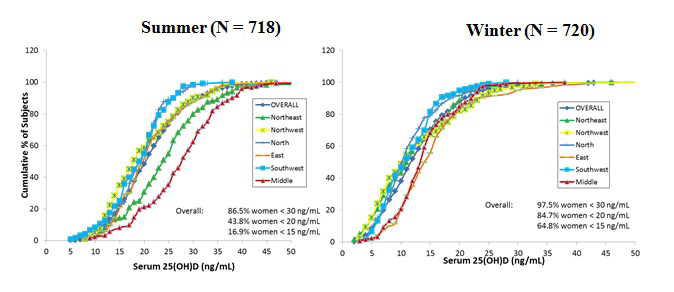

Supplement: Supplementary Figure 3 — Cumulative distribution for serum 25(OH)D overall and by region. Cumulative distribution curves of serum 25(OH)D levels by region and season (summer and winter) are displayed. Regional variation in the distribution of serum 25(OH)D was more distinct in the summer compared to the winter when the distributions for all regions were more similar to the overall distribution. Lower serum 25(OH)D was observed in the North and Southwest regions in both the summer and winter. [file Image_3.TIF]
